# Metabolic pathway engineering based on metabolomics confers acetic and formic acid tolerance to a recombinant xylose-fermenting strain of *Saccharomyces cerevisiae*

**DOI:** 10.1186/1475-2859-10-2

**Published:** 2011-01-10

**Authors:** Tomohisa Hasunuma, Tomoya Sanda, Ryosuke Yamada, Kazuya Yoshimura, Jun Ishii, Akihiko Kondo

**Affiliations:** 1 Organization of Advanced Science and Technology, Kobe University, 1-1 Rokkodai, Nada, Kobe, 657-8501, Japan; 2 Department of Chemical Science and Engineering, Graduate School of Engineering, Kobe University, 1-1 Rokkodai, Nada, Kobe 657-8501, Japan

## Abstract

**Background:**

The development of novel yeast strains with increased tolerance toward inhibitors in lignocellulosic hydrolysates is highly desirable for the production of bio-ethanol. Weak organic acids such as acetic and formic acids are necessarily released during the pretreatment (i.e. solubilization and hydrolysis) of lignocelluloses, which negatively affect microbial growth and ethanol production. However, since the mode of toxicity is complicated, genetic engineering strategies addressing yeast tolerance to weak organic acids have been rare. Thus, enhanced basic research is expected to identify target genes for improved weak acid tolerance.

**Results:**

In this study, the effect of acetic acid on xylose fermentation was analyzed by examining metabolite profiles in a recombinant xylose-fermenting strain of *Saccharomyces cerevisiae*. Metabolome analysis revealed that metabolites involved in the non-oxidative pentose phosphate pathway (PPP) [e.g. sedoheptulose-7-phosphate, ribulose-5-phosphate, ribose-5-phosphate and erythrose-4-phosphate] were significantly accumulated by the addition of acetate, indicating the possibility that acetic acid slows down the flux of the pathway. Accordingly, a gene encoding a PPP-related enzyme, transaldolase or transketolase, was overexpressed in the xylose-fermenting yeast, which successfully conferred increased ethanol productivity in the presence of acetic and formic acid.

**Conclusions:**

Our metabolomic approach revealed one of the molecular events underlying the response to acetic acid and focuses attention on the non-oxidative PPP as a target for metabolic engineering. An important challenge for metabolic engineering is identification of gene targets that have material importance. This study has demonstrated that metabolomics is a powerful tool to develop rational strategies to confer tolerance to stress through genetic engineering.

## Background

**Figure 1:**
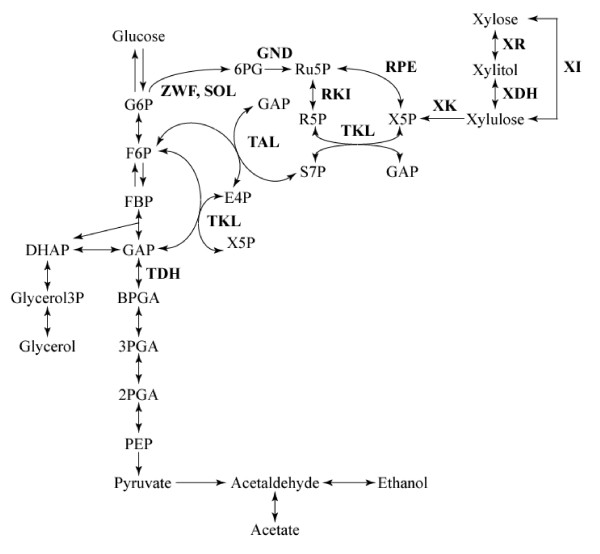
**Schematic representation of xylose metabolic pathway in metabolically engineered *S. cerevisiae *strains**. Abbreviations: BPGA, 1,3-*bis*phosphoglycerate; DHAP, dihydroxyacetonephosphate; E4P, erythrose-4-phosphate; FBP, fructose-1,6-*bis*phosphate; F6P, fructose-6-phosphate; GAP, glyceraldehyde-3-phosphate; Glycerol3P, glycerol-3-phosphate; G6P, glucose-6-phosphate; PEP, phospho*enol*pyruvate; 6PG, 6-phosphogluconate; 2PGA, 2-phosphoglycerate; 3PGA, 3-phosphoglycerate; R5P, ribose-5-phosphate; Ru5P, ribulose-5-phosphate; S7P, sedoheptulose-7-phosphate; X5P, xylulose-5-phosphate. Enzymes are indicated by their gene assignment symbols: GND, 6-phosphogluconate dehydrogenase; SOL, 6-phosphogluconolactonase; TAL, transaldolase; TDH, glyceraldehydes-3-phosphate dehydrogenase; TKL, transketolase; RPE, ribulose-5-phosphate 4-epimerase; RKI, ribose-5-phosphate isomerase; XI, xylose isomerase; XK, xylulokinase; XDH, xylitol dehydrogenase; XR, xylose reductase; ZWF, glucose-6-phosphate dehydrogenase.

Numerous environmental and social benefits could result from the replacement of petroleum-based transport fuels with bio-ethanol converted from lignocellulosic materials such as agricultural residues and industrial waste [1,2]. The commonly used yeast *Saccharomyces cerevisiae *has many advantages as an ethanol producer, such as fast sugar consumption, high ethanol yield from glucose, and higher resistance to ethanol and other compounds present in lignocellulosic hydrolysates than bacteria [3]. However, a major drawback is that *S. cerevisiae *cannot utilize xylose, the most common pentose sugar in the hemicellulose that makes up a sizable fraction of lignocellulosic hydrolysates. Thus, most efforts in the engineering of *S. cerevisiae *for xylose fermentation have focused on manipulation of the initial xylose metabolic pathway [4]. The reconstruction of an efficient xylose assimilation pathway in *S. cerevisiae *has been approached via heterologous expression of genes for xylose reductase (XR) and xylitol dehydrogenase (XDH) derived from *Pichia stipitis *along with overexpression of *S. cerevisiae *xylulokinase (XK) to produce ethanol in xylose fermentation [5-7]. Xylose is first reduced to xylitol by XR, and then xylitol is oxidized to xylulose by XDH. Xylulose is phosphorylated by XK to xylulose-5-phosphate (X5P), which is then metabolized through the non-oxidative pentose phosphate pathway (PPP) and the glycolysis pathway (Figure 1). On the other hand, a xylose isomerase (XI) gene derived from the anaerobic fungus *Piromyces *has also been introduced into *S. cerevisiae *[8]. XI converts xylose to xylulose in one step; however, the rate of xylose consumption is much lower in the XI-expressing strain [9].

To exploit lignocellulosic materials for fuel ethanol production, the improvement of not only fermentation capacity but also tolerance to compounds present in the hydrolysates is required. Contrary to sugarcane- or starch-derived feedstocks, lignocellulosic hydrolysates contain a variety of toxic compounds that negatively affect microbial growth, metabolism and ethanol yield. Harsh conditions used in the pretreatment of the raw material release inhibitors including weak organic acids, furan derivatives, and phenolics [10,11]. Specifically, the acetic acid that is released during solubilization and hydrolysis of hemicellulase [12] is usually found at a high concentration in the hydrolysate. Levels of acetate depend on the type of biomass and the pretreatment method. Concentrations typically range from 1 to 10 g/L in the hydrolysate [10]. Formic acid is typically present at lower concentrations than acetic acid, but is more toxic to *S. cerevisiae *than acetic acid [13,14]. Other toxic weak acids, for which the concentrations in the hydrolysate are rarely reported, are present at even lower concentrations than formic acid.

Although the mechanism of inhibition by weak acids is not easily elucidated, the inhibitory effect of weak acids has been ascribed to uncoupling and intracellular anion accumulation [10,12]. The undissociated form of weak acids can diffuse from the fermentation medium across the plasma membrane and dissociate due to higher intracellular pH, thus decreasing the cytosolic pH. In addition, intracellular accumulation of the anionic species may contribute to weak acid toxicity [15,16]. If the anionic form of acetic acid is captured in the cells, undissociated acid in the medium will diffuse into the cell until equilibrium is reached.

Improvement of yeast tolerance to lignocellulosic hydrolysates has been achieved by overexpressing homologous or heterologous genes encoding enzymes that confer resistance towards specific inhibitors such as furan derivatives and phenolic compounds [17-21]. In contrast, genetic engineering strategies that have addressed yeast tolerance to weak organic acids are rare [11]. Thus, enhanced basic research is expected to identify further target genes for improved acid tolerance.

The metabolic profiling technique [22] can be a powerful tool to gain insight into functional biology. The comprehensive analysis of a wide range of metabolites from cellular extracts with high-sensitivity mass spectrometry (MS) makes it well suited for the identification of metabolic compounds that are important for specific biological questions [23-25]. Metabolites are often the final downstream products or effects of gene expression and therefore can be used to assign or validate functional annotations related to enzyme activities in the associated metabolic pathway. Accordingly, such a metabolomic approach can be used for designing novel strategies to develop more efficient cell capacities through genetic engineering.

In the present study, the effect of acetic acid on xylose fermentation was determined through a metabolomic approach with both capillary electrophoresis-mass spectrometry (CE-MS) and gas chromatography-mass spectrometry (GC-MS), which revealed that the accumulation of metabolites involved in the non-oxidative PPP [e.g. sedoheptulose-7-phosphate (S7P), ribulose-5-phosphate (Ru5P), ribose-5-phosphate (R5P) and erythrose-4-phosphate (E4P)] increased with an increase in acetic acid concentration in a recombinant xylose-fermenting *S. cerevisiae *strain. Consequently, the gene encoding a PPP-related enzyme, transaldolase (TAL) or transketolase (TKL) (Figure 1) from *S. cerevisiae *was overexpressed in the strain, which conferred increased ethanol productivity in the presence of acetic and formic acids in the xylose-fermenting yeast.

## Results

### Effect of acetic acid on fermentation by a xylose-fermenting yeast

**Figure 2:**
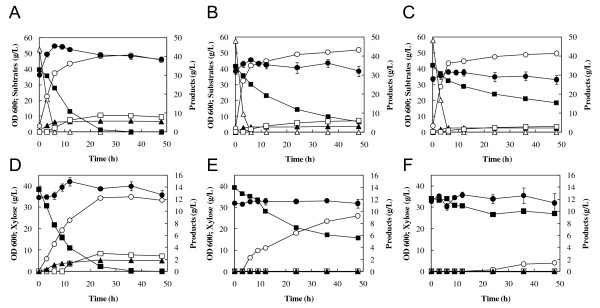
**Effects of acetic acid on the fermentation of glucose-xylose mixture (A-C) and xylose (D-F) at 30°C by *S. cerevisiae *MN8140X**. Acetic acid was added to the fermentation at a concentration of 0 mM (A, D), 30 mM (B, E) and 60 mM (C, F). Symbols: closed circles, biomass [optical density at 600 nm (OD 600)]; open triagnles, glucose; closed squares, xylose; open circles, ethanol; closed triangles, glycerol; open squares, xylitol. The values are the averages of four independent experiments, ± SEM.

To determine effects of acetic acid (30 or 60 mM) on glucose-xylose mixture fermentation by the XR/XDH/XK-based recombinant *S. cerevisiae *strain MN8140X, fermentation was performed anaerobically in YP medium containing 60 g/L glucose and 40 g/L xylose as carbon sources at 30°C after aerobic cultivation of cells in YPD medium. Initial cell concentration was adjusted to 50 g of wet cells/L. As shown in Figure 2A-C, the xylose consumption rate decreased with increase in acetic acid concentration, while glucose consumption remained nearly unaffected by the addition of 60 mM acetic acid. The specific xylose consumption rates of the strain were 0.36 ± 0.01, 0.19 ± 0.01 and 0.11 ± 0.01 g-xylose/g-cells/h at acetate concentrations of 0, 30 and 60 mM, respectively. Also, the amount of xylose consumed was decreased by the addition of acetic acid whereas glucose was completely consumed within 6 h even in 60 mM acetate. These results indicate that xylose fermentation by the XR/XDH/XK-based strain was more sensitive to acetic acid than glucose fermentation. In the absence of acetic acid, cell density observed at 600 nm was slightly increased after the initiation of the fermentation while the density was nearly unchanged during fermentation in the presence of 30 and 60 mM acetic acid. Subsequently, effects of acetic acid (30 or 60 mM) on the fermentation of xylose as a sole carbon source were determined. The fermentation was performed in YPX medium with or without acetic acid at 30°C using yeast cells aerobically prepared as described above; these cells also showed fermentation inhibition by acetic acid in a dose-dependent manner (Figure 2D-E). The addition of acetic acid caused reduction in the consumption of xylose and in the production of ethanol, xylitol and glycerol. After 24 h fermentation, OD 600 was 38.7 ± 0.8, 32.8 ± 0.8 and 33.9 ± 2.0 at acetate concentrations of 0 mM, 30 mM and 60 mM, respectively.

**Figure 3:**
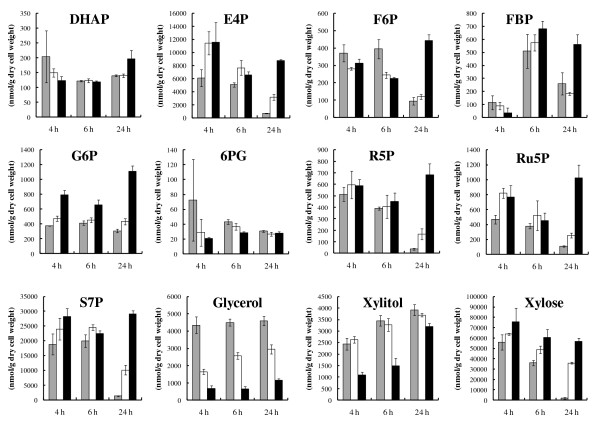
**Effects of acetic acid on the accumulation level of intracellular metabolites in xylose-fermenting *S. cerevisiae *MN8140X after fermentation for 4 h, 6 h, and 24 h**. Fermentation was carried out with 40 g/L xylose as a carbon source, and 0 mM (gray bars), 30 mM (white bars) or 60 mM (black bars) acetic acid at 30°C. The values are the averages of three independent experiments, ± SEM.

Thus, metabolome analysis of yeast cells that were used for the xylose fermentation was performed. Intracellular metabolites were extracted at 4, 6, and 24 h after the initiation of fermentation as described in Methods. Ionic metabolites such as sugar phosphates [dihydroxyacetonephosphate (DHAP), E4P, fructose-1,6-*bis*phosphate (FBP), fructose-6-phosphate (F6P), glucose-6-phosphate (G6P), 6-phosphogluconate (6PG), R5P, Ru5P and S7P], organic acids (fumarate, 2-ketoglutarate, malate, lactate, pyruvate and succinate), nucleotides (ADP and ATP) and coenzymes (CoA, acetyl-CoA, NAD^+^, NADH, NADP^+ ^and NADPH) were analyzed by CE-MS. As shown in Figure 3, in the absence of added acetic acid, non-oxidative PPP intermediates such as S7P, E4P, Ru5P and R5P decreased with time, whereas the decrease in these metabolites was slower in 30 mM acetate. Furthermore, on the addition of 60 mM acetic acid, the accumulation of these metabolites after 4 h fermentation did not decrease after 24 h. These results raise the possibility that the flux through the non-oxidative PPP in xylose fermentation was inhibited by the addition of acetate.

The decrease in R5P, Ru5P, S7P, E4P with time in the absence of acetate is likely due to the change of culture condition from aerobic to anaerobic cultivation. Before the initiation of xylose fermentation, these metabolites should be accumulated in the cells during the aerobic pre-cultivation in YPD medium. Figure 3 also shows increase of glycolytic intermediates such as DHAP, F6P, FBP and G6P by the addition of acetic acid. After 24 h of fermentation, 60 mM acetate condition caused the highest accumulation of the glycolytic metabolites. The accumulation levels of organic acids, nucleotides and coenzymes were shown in Additional file1

Sugars and sugar alcohols were quantified by GC-MS. The level of xylose in yeast cells clearly decreased with time in the absence of added acetic acid, while the level was almost constant in the presence of 60 mM acetate. In the case of 60 mM acetate, the intracellular content of xylitol and glycerol was lower than with 0 or 30 mM.

### Construction of strains expressing the *TAL1 *or *TKL1 *gene

In improving ethanol production in the presence of acetic acid, we focused on the excess accumulation of the non-oxidative PPP intermediates in yeast cells. In *S. cerevisiae *strains, TAL and TKL have been implicated as being rate-limiting steps for xylose fermentation [26]. TAL catalyzes the conversion of S7P and GAP to F6P and E4P, while TKL catalyzes the conversion of X5P and R5P to S7P and GAP, and the conversion of E4P and X5P to F6P and GAP (Figure 1). Thus, recombinant *S. cerevisiae *strains expressing the *TAL1 *gene coding TAL or the *TKL1 *gene coding TKL from *S. cerevisiae *under the control of the *PGK1 *promoter were constructed by homologous recombination (Table 1). The integration of these genes into the chromosomal genome of xylose-fermenting strain MT8-1X was confirmed by genomic PCR (data not shown). The activity of TAL in MT8-1X/TAL was 2.1-fold higher compared to the control strain (MT8-1X/pGK404) (Table 2). Also, the activity of TKL in MT8-1X/TKL was 3.1-fold higher compared to MT8-1X/pGK405.

**Table 1:** Characterization of *S. cerevisiae *strains and plasmids used in this study

Strain or plasmid	Description	Reference for source
*S. cerevisiae *strains		
MT8-1	*MAT*a *ade his3 leu2 trp1 ura3*	[49]
MT8-1X	MT8-1 (pIUX1X2XK)	[42]
MT8-1X/TAL	MT8-1X (pGK404ScTAL1)	This study
MT8-1X/pGK404	MT8-1X (pGK404)	This study
MT8-1X/TKL	MT8-1X (pGK405ScTKL1)	This study
MT8-1X/pGK405	MT8-1X (pGK405)	This study
NBRC1440ΔHUWL	*MAT*α *his3 leu2 trp1 ura3*	[50]
NBRC1440X	NBRC1440 ΔHUWL (pIUX1X2XK)	This study
MT8-1X/pRS403	MT8-1X (pRS403)	This study
MN8140X	*MAT*a/α *leu2 trp1 *(pIUX1X2XK)	This study
Plasmids		
pIUX1X2XK	*URA3*, expression of *XYL1*, *XYL2 *and *XKS1 *genes	[42]
pGK404ScTAL1	*HIS3*, expression of *S. cerevisiae TAL1 *gene	This study
pGK405ScTKL1	*LEU2*, expression of *S. cerevisiae TKL1 *gene	This study
pGK404	*HIS3*, no expression (control plasmid)	[41]
pGK405	*LEU2*, no expression (control plasmid)	[41]
pRS403	*HIS3*, no expression (control plasmid)	Stratagene

**Table 2:** *In vitro *enzyme activities of TAL and TKL in recombinant *S. cerevisiae *strains. *^a^*

	Specific activity (U/mg protein)	
	
Strain	TAL	TKL
MT8-1X/TAL	0.111 ± 0.009	0.066 ± 0.021
MT8-1X/pGK404	0.054 ± 0.004	0.052 ± 0.016
MT8-1X/TKL	0.027 ± 0.011	0.149 ± 0.009
MT8-1X/pGK405	0.021 ± 0.012	0.048 ± 0.013

*^a ^*The values are the averages of three independent experiments, ±SEM.

### The effect of acetic acid on xylose-fermenting strains expressing the *TAL1 *or *TKL1 *gene

**Figure 4:**
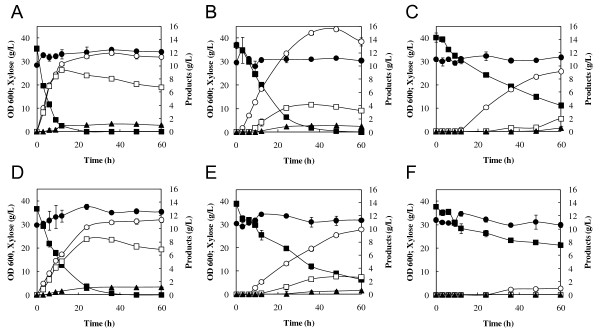
**Effects of acetic acid on fermentation of xylose as a sole carbon source at 30°C using *S. cerevisiae *MT8-1X/TAL (A-C) and MT8-1X/pGK404 (D-F)**. Acetic acid was added to the fermentation at a concentration of 0 mM (A, D), 30 mM (B, E) of 60 mM (C, F). The values are the averages of four independent experiments, ± SEM. Symbols: close circles, biomass (OD 600); closed squares, xylose; open circles, ethanol; closed triangles, glycerol; open squares, xylitol.

The recombinant strain MT8-1X/TAL was used for xylose fermentation with or without addition of acetic acid (Figure 4). MT8-1X/TAL showed a higher ethanol production rate and a higher xylose consumption rate than the control strain, regardless of acetic acid concentration. The specific xylose consumption rates of MT8-1X/TAL were 0.36 ± 0.01, 0.20 ± 0.01 and 0.12 ± 0.01 g-xylose/g-cells/h at acetate concentrations of 0, 30 and 60 mM, respectively, while those of the control strain were 0.26 ± 0.01, 0.13 ± 0.02 and 0.10 ± 0.02 g-xylose/g-cells/h. The specific ethanol productivities of MT8-1X/TAL was 0.04 ± 0.00, 0.09 ± 0.00 and 0.03 ± 0.00 g-ethanol/g-cells/h at acetate concentrations of 0, 30 and 60 mM, respectively, while those of the control strain was 0.03 ± 0.00, 0.03 ± 0.00 and 0.00 ± 0.00 g-ethanol/g-cells/h. In the presence of 30 mM acetic acid, ethanol concentration in MT8-1X/TAL was 1.72-fold higher than that in MT8-1X/pGK404 after 48 h of fermentation (Table 3). The yield (in grams of ethanol produced per gram of xylose consumed) increased from 0.301 g/g in the control strain to 0.425 g/g in MT8-1X/TAL. Interestingly, ethanol concentration (15.55 g/L) in MT8-1X/TAL in the presence of 30 mM acetic acid was 1.35-fold higher than that (11.50 g/L) in the absence of acetic acid. MT8-1X/TAL produced 8.33 g/L of ethanol in 60 mM acetate, while almost no ethanol was observed in the control after 48 h of fermentation. These results indicate that the overexpression of the *TAL1 *gene improved ethanol fermentative capacity in the presence of acetic acid in the xylose-fermenting yeast. The content of the non-oxidative PPP intermediates such as S7P, R5P and Ru5P was decreased by the expression of the *TAL1 *gene (Table 4). In the presence of 60 mM acetic acid, after 24 h of fermentation, the content of S7P, R5P and Ru5P in MT8-1X/TAL was 6.4-, 3.1- and 1.8-fold lower than that in the control strain. In both MT8-1X/TAL and MT8-1X/pGK404 strains, xylitol production was decreased by the addition of acetate. The concentrations of acetate added were constant during the fermentation (data not shown).

**Table 3:** Ethanol concentration, ethanol yield, xylitol yield, and glycerol yield after 48 h of fermentation of 40 g/L xylose with different concentrations of acetic acid by recombinant *S. cerevisiae *strains. *^a^*

Strain	Acetic acid	Ethanol (g/L)	**Y **_**(Ethanol/Xylose) **_**(g/g)**	**Y**_**(Xylitol/Xylose) **_**(g/g)**	**Y**_**(Glycerol/Xylose) **_**(g/g)**
MT8-1X/TAL	0 mM	11.50 ± 0.41	0.324 ± 0.013	0.203 ± 0.011	0.032 ± 0.002
	30 mM	15.55 ± 0.01	0.425 ± 0.018	0.105 ± 0.005	0.027 ± 0.002
	60 mM	8.33 ± 0.27	0.328 ± 0.025	0.021 ± 0.024	0.003 ± 0.004
MT8-1X/pGK404	0 mM	11.13 ± 0.40	0.304 ± 0.011	0.200 ± 0.011	0.031 ± 0.001
	30 mM	9.05 ± 0.11	0.301 ± 0.010	0.090 ± 0.004	0.015 ± 0.001
	60 mM	0.88 ± 0.31	0.053 ± 0.022	0 ± 0	0 ± 0

*^a ^*The values are the averages of four independent experiments, ±SEM.

**Table 4:** Content of PPP intermediates in recombinant strains under different acetate condition. *^a^*

	Metabolite content (nmol/g dry cell weight) *^b^*			
	
	MT8-1X/TAL		MT8-1X/pGK404	
	
	0 mM Acetate	60 mM Acetate	0 mM Acetate	60 mM Acetate
6PG	18.89 ± 0.58	24.87 ± 1.99	77.71 ± 16.09	33.23 ± 3.77
R5P	14.59 ± 7.64	130.48 ± 10.56	230.12 ± 34.22	409.51 ± 79.03
Ru5P	24.20 ± 5.70	201.70 ± 16.05	217.88 ± 22.98	367.92 ± 72.41
S7P	657.80 ± 8.37	986.61 ± 41.26	3270.01 ± 448.68	6317.95 ± 641.64

*^a ^*The values are the averages of three independent experiments, ±SEM.

*^b ^*Metabolites were extracted from recombinant *S. cerevisiae *strains after 24 h fermentation of 40 g/l xylose at 30°C.

**Figure 5:**
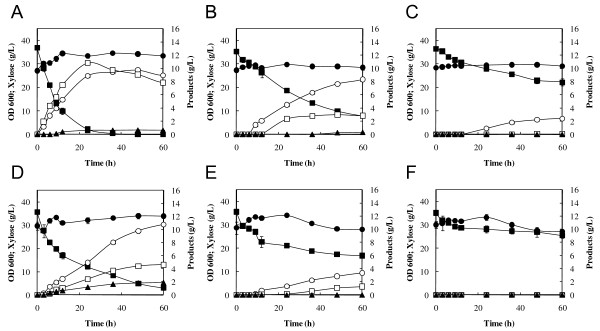
**Effects of acetic acid on fermentation of xylose as a sole carbon source at 30°C using *S. cerevisiae *MT8-1X/TKL (A-C) and MT8-1X/pGK405 (D-F)**. Acetic acid was added to the fermentation at a concentration of 0 mM (A, D), 30 mM (B, E) or 60 mM (C, F). The values are the averages of four independent experiments, ±SEM. Symbols: closed circles, biomass (OD 600); closed squares, xylose; open circles, ethanol; closed triangles, glycerol; open squares, xylitol.

The effect of expression of the *TKL1 *gene on xylose fermentation in the presence of acetic acid was also determined (Figure 5). The recombinant strain MT8-1X/TKL showed a higher ethanol production rate and a higher xylose consumption rate compared to the control strain (MT8-1X/pGK405) in xylose fermentation with or without acetic acid. The ethanol concentration in the presence of acetic acid (30 and 60 mM) was also increased by the expression of the *TKL1 *gene. However, the impact of *TKL1 *expression on acetate tolerance was smaller than that of *TAL1 *expression. Also, a recombinant strain that expresses both the *TAL1 *and *TKL1 *genes under the control of the *PGK1 *promoter was constructed, which showed no increase in ethanol production from xylose in the presence of acetic acid compared to the control strain (data not shown).

The addition of acetic acid slightly inhibited cell growth of MT8-1X/TAL and MT8-1X/TKL as it did the control strains. However, ethanol fermentation ability was improved by the expression of *TAL1 *and *TKL1*. These results support ethanol production in the presence of acetic acid being improved by the alteration in xylose metabolism.

### Effect of formic acid on xylose-fermenting strain expressing the *TAL1 *gene

**Figure 6:**
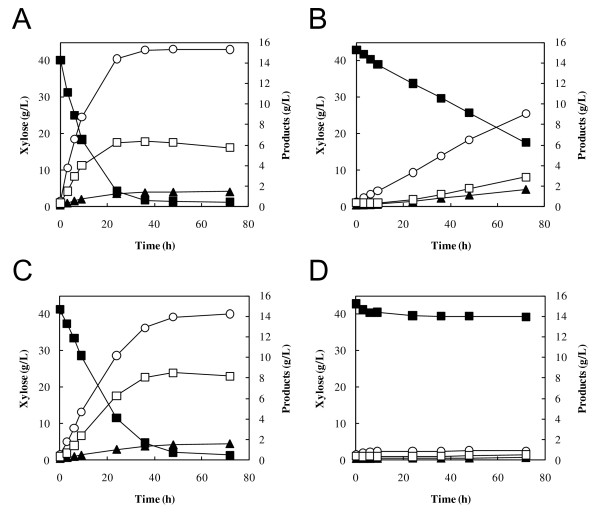
**Effects of formic acid on fermentation of xylose as a sole carbon source using *S. cerevisiae *MT8-1X/TAL (A, B) and MT8-1X/pGK404 (C, D)**. Formic acid was added to the fermentation at a concentration of 15 mM (A, C) or 30 mM (B, D). Symbols: closed squares, xylose; open circles, ethanol; closed triangles, glycerol; open squares, xylitol.

Formic acid has a more inhibitory effect on the yield of ethanol in glucose fermentation than acetic acid [27]. The increased toxicity seems to be associated with its high permeability through the plasma membrane [10]. In the present study, MT8-1X/TAL1 demonstrated higher ethanol production in the presence of 15 mM and 30 mM formic acid compared with the control strain (Figure 6).

## Discussion

We demonstrated that acetic acid negatively affected the non-oxidative PPP in xylose-fermenting *S. cerevisiae *strains by the determination of comprehensive metabolic intermediates through a metabolomic approach. Subsequently, the overexpression of the *TAL1 *gene encoding transaldolase, which is assumed to be one of the rate-limiting enzymes in the non-oxidative PPP, successfully reduce the inhibition of the fermentation capacity by weak acids such as acetic and formic acid. Our results demonstrated that metabolomics is a powerful tool to gain insight into the effects of environmental perturbation on microbial metabolism and then to develop rational strategies to confer tolerance to the stress through genetic engineering.

As shown in Figure 2, the xylose consumption rate was more severely affected by the addition of acetic acid than the glucose consumption rate. This specific effect of acetic acid on the fermentation of xylose has been observed with strains based on the expression of xylose isomerase (XI) from *Piromyces *[28]. In a previous report [29], ethanol yield on xylose was more sensitive to acetic acid than the yield on glucose in fermentation with a glucose-xylose mixture by an XR/XDH-based recombinant strain. In genetically engineered *S. cerevisiae *strains, xylulose formed by XR/XDH or XI from xylose is phosphorylated to X5P and channeled via the non-oxidative PPP into glycolysis (Figure 1). Although glucose was metabolized via glycolysis or PPP, a large part of glucose would be converted to ethanol through glycolysis rather than PPP during anaerobic fermentation according to metabolic flux analysis [30]. Our results raise the possibility that the xylose-specific effect is caused by the inhibition of the xylose metabolic pathway such as the non-oxidative PPP or xylose uptake through transporter. In fact, this possibility was supported by the accumulation of metabolic intermediates involved in the non-oxidative PPP (Figure 3).

As shown in Figure 2, cell density slightly increased in the absence of acetic acid while it was constant during fermentation in the presence of acetic acid. These results indicate that acetic acid inhibits cell growth. On the other hand, there was no difference in cell density between 30 mM and 60 mM acetate although the levels of intracellular metabolites altered by the increased acetic acid. Therefore, acetic acid would negatively affect xylose metabolism.

TAL and TKL have been considered rate-limiting enzymes in the non-oxidative PPP, which is supported by several genome-scale and enzymatic analyses of mutant strains with improved xylose metabolism, in which *TAL1*, *TKL1*, or both genes have been found to be upregulated [30-34]. We revealed that the overexpression of *TAL1 *or *TKL1 *genes in recombinant xylose-fermenting strains improved ethanol production in the presence of acetic acid. In the presence of 30 mM or 60 mM acetic acid, the ethanol concentration in the *TAL1*-expressing strain was 1.72-fold or 9.47-fold higher than that in the control strain after 48 h of fermentation (Table 3). The increase in the ethanol yield by the addition of 30 mM acetic acid in the strain might be correlated with the decrease in the xylitol yield. So far, metabolic engineering studies to improve the tolerance of *S. cerevisiae *to weak organic acids have been rare [11]. Success in different backgrounds, for instance an acetate-tolerant *S. cerevisiae *strain selected for use in sourdough [35], could be extrapolated to glucose fermentation in the presence of weak acids. To our knowledge, however, XR/XDH/XK-based recombinant strains have not been reported to have acquired tolerance to weak organic acids through metabolic engineering.

MT8-1X/TAL showed the highest ethanol concentration when it was exposed to 30 mM acetic acid (Table 3). A previous fermentation of a softwood (spruce) hydrolysate solution mainly containing glucose indicated a similar trend that low acid concentrations (< 100 mM) increased the ethanol yield at pH 5.5, whereas the yield decreased at higher concentration [27]. It is postulated that a low concentration of acid stimulates the production of ATP, which is achieved under anaerobic conditions by ethanol production [10]. The decrease in intracellular pH by uptake of undissociated acetic acid could be neutralized by the plasma membrane ATPase, which pumps protons out of the cell at the expense of ATP hydrolysis. Additional ATP must be generated in order to maintain the intracellular pH.

The ethanol production from xylose with acetic acid in MT8-1X/TKL was significantly less than that in MT8-1X/TAL (Figures 4 &5), indicating that the enhancement of *TAL1 *expression is more effective than that of *TKL1 *expression for the improvement of tolerance to acetic acid. This might be due to the increase in the flux of the non-oxidative PPP by TAL. An early attempt to overexpress *P. stipitis TKL *in xylose-metabolizing *S. cerevisiae *was unsuccessful [36], whereas overexpression of the endogenous *S. cerevisiae TAL1 *resulted in improved growth on xylose [37]. In our study, co-expression of the *TAL1 *and the *TKL1 *genes in MT8-1X did not lead to an increase in the ethanol production from xylose in the presence of acetic acid compared to the control strain. The expression of only *TAL1 *gene might be sufficient to relieve the negative regulation of the non-oxidative PPP by acetic acid. Possibly, optimization of the ratio of overexpressed enzymes in the non-oxidative PPP should be considered in future studies.

The biochemical and molecular basis of yeast stress tolerance has been investigated by a global gene expression analysis using a DNA microarray [38]. Evolutionarily engineered yeast that tolerates furfural and 5-hydroxymethyfurfural showed enhanced expression of *ZWF1*, *GND1*, *GND2*, and *TDH1*, genes involved in NAD(P)H regeneration steps in PPP and glycolysis pathway [39]. Also, screening of single gene deletion mutant library revealed that PPP genes *ZWF1*, *GND1*, *RPE1*, and *TKL1 *were required for furfural tolerance in *S. cerevisiae *[17]. These reports support PPP being intimately associated with yeast stress tolerance to fermentation inhibitors.

As shown in Figure 3, the addition of acetic acid to the xylose fermentation led to the accumulation of intracellular xylose, while the xylitol level reduced by the addition of 60 mM acetate. The xylitol content in the fermentation medium also decreased (Figure 2). These results raise the possibility that the activity of XR is negatively affected by the addition of acetic acid.

The inhibition of yeast fermentation by weak acid is clearly a significant hurdle for the development of ethanol production from lignocellulose. However, there are only a few reported studies on strategies to overcome this inhibition. During the past few years, global gene expression analysis and proteomic analysis have been performed under conditions mimicking the authentic ethanol fermentation process, presenting dynamic views of gene expression and protein biosynthesis in the yeast stress response [38]. As a part of systems biology, metabolomics represents the distal read-out of cellular status in contrast to genomics and proteomics, and more closely reflects the activities of the cell at a functional level. We report herein a successful application of metabolomics to breeding robust *S. cerevisiae *strains that have higher stress tolerance and ethanol productivity. An important challenge for metabolic engineering is identification of gene targets that have material importance for improvement of cellular function. This study supports metabolomics as facilitating metabolic engineering to optimize microorganisms for industrial ethanol production from lignocellulosic feedstocks through white biotechnology.

## Conclusions

Our metabolomic approach revealed that metabolic intermediates involved in the non-oxidative PPP were accumulated in xylose-fermenting *S. cerevisiae *strains by the addition of 30-60 mM acetic acid to cause reduction in xylose consumption and ethanol production, raising the possibility that the metabolism of the non-oxidative PPP was inhibited by the addition of acetic acid. Accordingly, a recombinant strain overexpressing *TAL1 *gene was constructed, which successfully improved ethanol production from xylose in the presence of acetic acid with decrease in the accumulation of the PPP intermediates. The *TAL1*-expressing strain also demonstrated higher ethanol production in the presence of 15-30 mM formic acid. The present work has demonstrated that metabolomics is a powerful tool to gain insight into the effects of environmental perturbation on microbial metabolism and then to design rational strategies to confer tolerance to the stress through metabolic engineering.

## Methods

### Microbial strains and media

Microbial strains used in this study are listed in Table 1. Yeast strains were routinely cultivated at 30°C in synthetic medium [SD medium; 6.7 g/L of yeast nitrogen base without amino acids (Difco Laboratories, Detroit, MI), 20 g/L of glucose] supplemented with appropriate amino acids and nucleotides, and in YPD medium (20 g/L peptone, 10 g/L yeast extract, 20 g/L glucose). *Escherichia coli *NovaBlue (Novagen, Inc., Madison, WI) was used as the host strain for recombinant DNA manipulation. *E. coli *was grown in Luria-Bertani medium (10 g/L peptone, 5 g/L yeast extract, and 5 g/L sodium chloride) containing 100 mg/L ampicillin.

### Construction of plasmids

Plasmids used in this study are listed in Table 1. Standard techniques for nucleic acid manipulation were used as described by Sambrook et al. [40]. The *TAL1 *gene, encoding TAL protein, was amplified using genomic DNA of *S. cerevisiae *strain MT8-1 as a template and the primer set P1 (5'-ATCAGGACTAGTATGTCTGAACCAGCTCAAAAG-3')/P2 (5'-AATCGCGGATCCTTAAGCGGTAACTTTCTTTTCAATC-3'). The *Spe*I and *Bam*HI sites are underlined. Similarly, the *TKL1 *gene, encoding TKL protein, was amplified from the same genomic DNA using the primer set P3 (5'-ACGCGTCGACATGACTCAATTCACTGACATTG-3')/P4 (5'-ATCAGGACTAGTTTAGAAAGCTTTTTTCAAAGGAG-3'). The *Sal*I and *Spe*I sites are underlined. PCR-amplified *TAL1 *gene was digested with *Spe*I and *Bam*HI, then ligated into the *Spe*I-*Bam*HI site of pGK404 [41] to yield plasmid pGK404ScTAL1. Similarly, *TKL1 *gene was digested with *Sal*I and *Spe*I, then ligated into the *Sal*I-*Spe*I site of pGK405 [41] to yield plasmid pGK405ScTKL1.

### Yeast transformation

Yeast transformants constructed in this study are summarized in Table 1. Transformation was performed by the lithium-acetate method, using the Yeastmaker transformation system (Clontech Laboratories, Inc., Palo Alto, CA). Transformants were selected on SD medium supplemented with appropriate amino acids and nucleotides. The plasmids pGK404, pGK404ScTAL1, pGK405, and pGK405ScTKL1 were digested with *Eco*RV and the linear products were transformed into *S. cerevisiae *strain MT8-1X. The plasmids pRS403 (Stratagene, La Jolla, CA) was digested with *Msc*I and the linearized product was transformed into the strain MT8-1X to yield MT8-1X/pRS403. Similarly, pIUX1X2XK [42] was digested with *Pst*I and transformed NBRC1440ΔHUWL to yield NBRC1440X.

### Mating

Diploid strain MN8140X was constructed by mating haploid strains MT8-1X/pRS403 and NBRC1440X. Individual haploid strains grown on YPD liquid medium for 24 h were harvested and spread together on YPD plates. After incubation for 72 h at 30°C, resultant strain was replica-plated to SD plates, and incubated for 3 days at 30°C. The resulting diploid strain formed single colonies on SD plates.

### Fermentation

The yeast transformants were aerobically cultivated in YPD medium for 48 h at 30°C. Cells were collected by centrifugation at 1,000 × *g *for 5 min at 4°C and washed twice with distilled water. The cells were inoculated into 50 ml of fermentation medium (YPX medium: 10 g/L yeast extract, 20 g/L peptone, and 40 g/L xylose, or YPDX medium: 10 g/L yeast extract, 20 g/L peptone, 60 g/L glucose, and 40 g/L xylose) containing acetic acid. All fermentations were performed at 30°C with mild agitation in 100 ml closed bottles equipped with a bubbling CO_2 _outlet. The initial cell concentration was adjusted to 50 g of wet cells/L. Wet cell weight was determined by weighing a cell pellet that was harvested by centrifugation at 1,000 × *g *for 5 min. The estimated dry cell weight was approximately 0.15-fold the wet cell weight. For determination of the concentrations of ethanol, glucose, glycerol, xylitol and xylose in the fermentation medium, the supernatant obtained by centrifugation at 14,000 × *g *at 4°C for 5 min was applied to high performance liquid chromatograph (HPLC) (Shimadzu, Kyoto, Japan) equipped with a Shim-pack SPR-Pb column (7.8 mm × 250 mm; Shimadzu) and an RID-10A refractive index detector (Shimadzu). The HPLC system was operated at 80°C, with water at a flow rate of 0.6 ml/min as the mobile phase. Cell growth was monitored via optical density at 600 nm on a UVmini-1240 spectrophotomer (Shimadzu).

### Enzyme assays

Cells from 50 ml samples of steady-state cultures in YPD medium were harvested by centrifugation at 5,000 × *g *at 4°C for 5 min. The pellet was washed once with 10 mM potassium phosphate buffer (pH 7.5) containing 2 mM EDTA, and suspended in 100 mM potassium phosphate buffer (pH 7.5) containing 2 mM MgCl_2 _and 2 mM DTT. The suspended cells were mixed with glass beads (0.5 mm diameter), disrupted by shaking at 2,500 rpm at 4°C for 5 min with a Multi-beads shocker (Yasui Kikai Corporation, Osaka, Japan). The crude extract, collected after centrifugation at 30,000 × *g *at 4°C for 30 min, was used for the enzyme assay. TAL activity was measured by monitoring NADH oxidation at 340 nm as described previously [43]. TKL activity was measured according to the method of Bruinenberg et al [44], except that 100 mM triethanolamine buffer (pH 7.8) was used as the buffer. The specific activities of the enzymes were expressed as micromoles of converted substrate per milligram of protein per minute (which is equivalent to units per milligram). Protein concentrations were determined by using a protein assay kit (Bio-Rad, Hercules, CA), with bovine serum albumin as the standard.

### Extraction of intracellular metabolites

Samples for metabolome analysis were prepared as described previously [45,46]. Leakage-free quenching was performed according to Canelas et al. [46]. Briefly, 5 ml of broth was withdrawn and injected (< 1 s) into a tube containing 7 ml of pure methanol pre-cooled to -40°C, the mixture was quickly vortexed and placed back in the cryostat (-40°C). Extracellular medium was removed by centrifugation at 5,000 × *g *at -20°C for 5 min. After decanting, 7.5 μl of 1 mM 1,4-piperazinediethanesulfonic acid (PIPES) and 7.5 μl of 100 mM adipic acid was added to the samples as internal standards. PIPES was used as an internal standard in CE-MS as described in a previous report [47]. For GC-MS, since the levels of measured metabolites were higher than that of metabolites measured by CE-MS, adipic acid was used as another internal standard. Metabolites were extracted by boiling ethanol method as described previously [45]. 75% (v/v) ethanol preheated in a water bath at 95°C was quickly poured over the cell pellet; the mixture was immediately vortexed, and the sample was placed in the water bath for 3 min. The extracts were evaporated under vacuum using a Freeze Dry System, FreeZone 2.5 Plus (Labconco, Kansas City, MO). Dried residues of samples were stored at -80°C until further use.

### Metabolite analysis with GC-MS

Dried metabolites were derivatized at 30°C for 90 min with 50 μl of 20 mg/ml methoxyamine hydrochloride in pyridine, and at 37°C for 30 min after the addition of 100 μl of *N*-methyl-*N*-(trimethylsilyl)trifluoroacetamide (GL Science, Tokyo, Japan) as described in a previous paper [48]. The derivatized sample (1 μl) was injected via an Agilent 7683B autosampler into an Agilent 7890A gas chromatograph (Agilent Technologies, Palo Alto, CA) equipped with a 30 m × 0.25 mm i.d. fused-silica capillary column coated with 0.25-μm CP-Sil 8 CB low bleed (Varian Inc., Palo Alto, CA) and coupled to a Pegasus HT time of flight mass spectrometer (TOFMS) (Leco Corp., St Joseph, MI). The injector temperature was 230°C and the helium gas flow rate through the column was 1 ml/min. The column temperature was held at 80°C for 2 min, then raised by 15°C/min to 330°C, and held there for 10 min. The transfer line and the ion source temperature were set at 250°C and 200°C, respectively. The ion source was generated by a 70 eV electron beam; 20 scans per second were recorded in the mass range of 85 to 500 *m/z*. The acceleration voltage was turned on after a solvent delay of 200 s. Peak deconvolution identification and quantification were performed using the Pegasus ChromaTOF software package ver 4.21 (Leco). Commercially available standard compounds were derivatized and analyzed in parallel with the samples. The mass spectra and retention time obtained were used to identify the metabolites. Unique fragment ions were used for peak area calculation.

### Metabolite analysis with CE-MS

Dried metabolites were dissolved in 15 μl of Milli-Q water before CE-MS analysis. All CE-MS experiments were performed using an Agilent CE capillary electrophoresis system, an Agilent G6220AA LC/MSD TOF system, and an Agilent 1200 series isocratic HPLC pump. For system control and data acquisition, Agilent ChemStation software for CE and MassHunter software for the Agilent TOFMS were operated. CE separation was performed in a fused silica capillary (1 m × 50 μm i.d.) filled with 30 mM ammonium formate (pH 10.0) as electrolyte. Prior to the first use, each capillary was washed with running electrolyte for 20 min with application of 50 mbar pressure. Before injection in each analysis, the capillary was equilibrated for 2 min with 1 M formic acid, then for 4 min with preconditioning buffer (15 mM ammonium acetate, 75 mM sodium phosphate, pH 7.5), 4 min with water, and 6 min with electrolyte, which was replenished every run using a buffer replenishment system equipped with the Agilent CE. Sample was injected with a pressure of 50 mbar for 10 or 30 s (approximately 10 or 30 nL). The CE polarity was such that the electrolyte vial (inlet) was at the anode, and the ESI probe (outlet) was at the cathode. The applied voltage to the CE capillary was set at 30 kV with 0.3 min of ramp time. Electrophoresis was performed for 50 min. The capillary temperature was maintained at 20°C and the sample tray was cooled below 6°C. An HPLC pump equipped with 1:100 splitter was used to deliver 8 μl/min of 50% (v/v) methanol/water containing reference masses of the standards purine ([M-H]^-^, *m/z *119.03632) and hexakis(1H,1H,3H-tetrafluoroproxy)phosphazine ([M-H]^-^, *m/z *966.000725) to the CE interface where it was used as a sheath fluid around the outside of the CE capillary to provide a stable electrical connection between the tip of the capillary and grounded electrospray needle. ESI-MS was conducted in the negative ion mode and the capillary voltage was set at 3.5 kV. The nebulizing gas, heated dry nitrogen (heater temperature 300°C), was switched off during the preconditioning step, and a pressure of 10 psi was applied 1 min after sample injection using the CE system's timetable.

## Abbreviations

BPGA: 1,3-*bis*phosphoglycerate; CE-MS: capillary electrophoresis-mass spectrometry; DHAP: dihydroxyacetonephosphate; E4P: erythrose-4-phosphate; FBP: fructose-1,6-*bis*phosphate; F6P: fructose-6-phosphate; GAP: glyceraldehyde-3-phosphate; GC-MS: gas chromatography-mass spectrometry; Glycerol3P: glycerol-3-phosphate; GND: 6-phosphogluconate dehydrogenase; G6P: glucose-6-phosphate; PEP: phospho*enol*pyruvate; 6PG: 6-phosphogluconate; 2PGA: 2-phosphoglycerate; 3PGA: 3-phosphoglycerate; PIPES: 1,4-piperazinediethanesulfonic acid; PPP: pentose phosphate pathway; RKI: ribose-5-phosphate isomerase; R5P: ribose-5-phosphate; RPE: ribulose-5-phosphate 4-epimerase; Ru5P: ribulose-5-phosphate; SOL: 6-phosphogluconolactonase; S7P: sedoheptulose-7-phosphate; TAL: transaldolase; TDH: glyceraldehydes-3-phosphate dehydrogenase; TKL: transketolase; XDH: xylitol dehydrogenase; XI: xylose isomerase; XK: xylulokinase; X5P: xylulose-5-phosphate; XR: xylose reductase; ZWF: glucose-6-phosphate dehydrogenase.

## Competing interests

The authors declare that they have no competing interests.

## Authors' contributions

TH designed research and wrote the manuscript. TS performed yeast transformation, fermentation and metabolite analysis. RY performed yeast transformation. KY performed enzyme assay and metabolite analysis. JI performed plasmid construction and commented on the manuscript. AK commented and supervised on the manuscript. All authors have read and approved the manuscript.

## Supplementary Material

Additional file 1 **Effects of acetic acid on the accumulation level of intracellular metabolites in xylose-fermenting *S. cerevisiae *MN8140X after fermentation for 4 h, 6 h, and 24 h**. Fermentation was carried out with 40 g/L xylose as a carbon source, and 0 mM (gray bars), 30 mM (white bars) or 60 mM (black bars) acetic acid at 30°C. The values are the averages of three independent experiments, ± SEM. Click here for file
