# Supplementary figures and images for: Metabolic pathway engineering based on metabolomics confers acetic and formic acid tolerance to a recombinant xylose-fermenting strain of Saccharomyces cerevisiae
Source: Microb Cell Fact. 2011 Jan 10;10:2. doi: 10.1186/1475-2859-10-2 (PMC3025834; doi:10.1186/1475-2859-10-2)

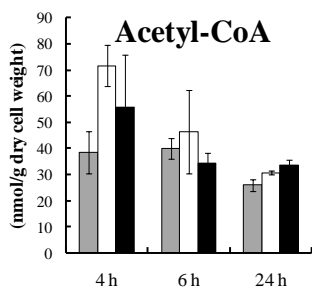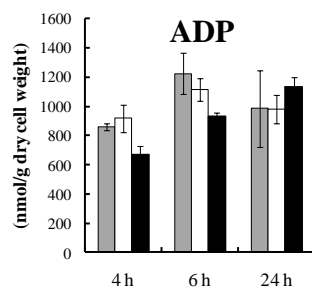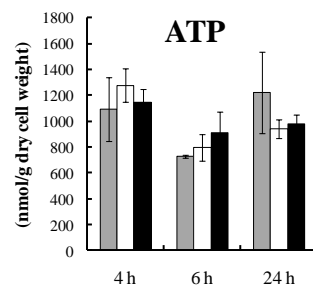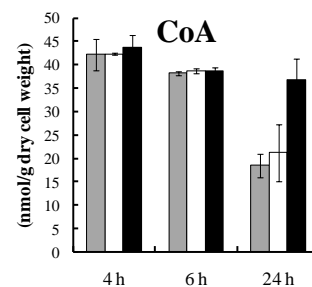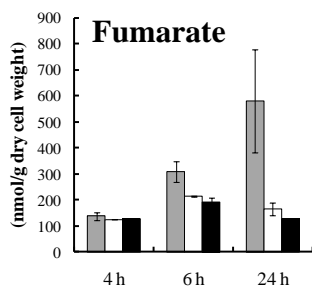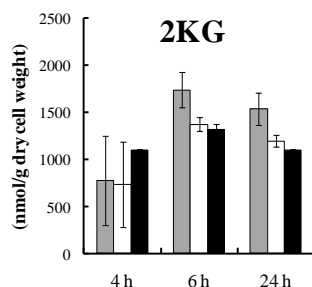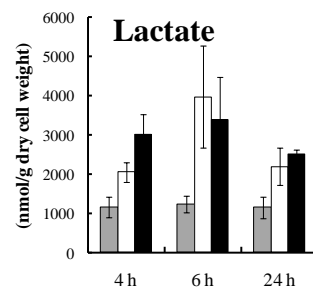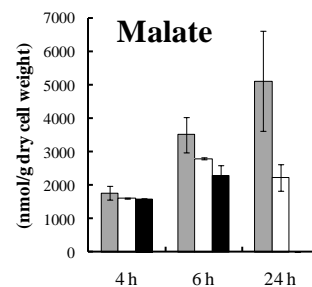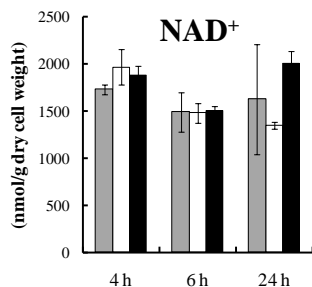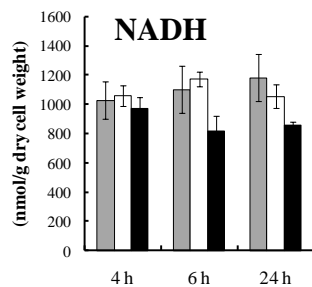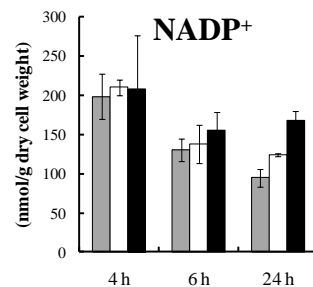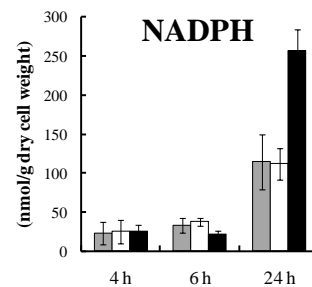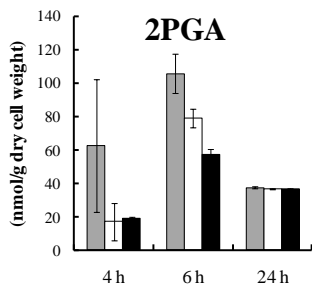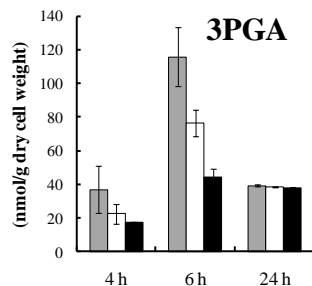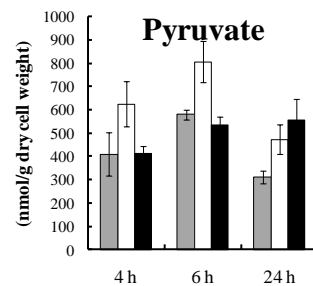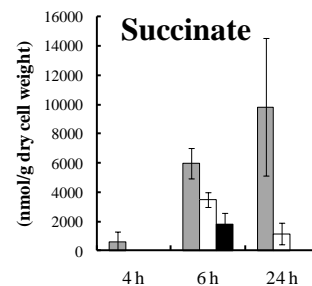

Supplement: Additional file 1 — Effects of acetic acid on the accumulation level of intracellular metabolites in xylose-fermenting S. cerevisiae MN8140X after fermentation for 4 h, 6 h, and 24 h. Fermentation was carried out with 40 g/L xylose as a carbon source, and 0 mM (gray bars), 30 mM (white bars) or 60 mM (black bars) acetic acid at 30°C. The values are the averages of three independent experiments, ± SEM. [file 1475-2859-10-2-S1.PDF]
